# *Halophilomyces hongkongensis*, a Novel Species and Genus in the Lulworthiaceae with Antibacterial Potential, Colonizing the Roots and Rhizomes of the Seagrass *Halophila ovalis*

**DOI:** 10.3390/jof10070474

**Published:** 2024-07-10

**Authors:** Xiao Wang, Lorenzo Pecoraro, Jiawei Chen, Yang Tang, Sangwook Lee, Sheng Chen, Hongbin Liu

**Affiliations:** 1 Department of Ocean Science, The Hong Kong University of Science and Technology, Hong Kong; xwanghw@connect.ust.hk (X.W.);; 2 College of Pharmaceutical Science & Moganshan Research Institute at Deqing County, Zhejiang University of Technology, Hangzhou 310014, China; 3 School of Pharmaceutical Science and Technology, Tianjin University, 92 Weijin Road, Tianjin 300072, China; 4 Department of Infectious Diseases and Public Health, Jockey Club College of Veterinary Medicine and Life Sciences, City University of Hong Kong, Hong Kong; 5 State Key Laboratory of Chemical Biology and Drug Discovery, Department of Food Science and Nutrition, The Hong Kong Polytechnic University, Hong Kong

**Keywords:** Lulworthiales, marine fungi, seagrass, novel taxa, phylogeny, antibacterial activity

## Abstract

Seagrass serves as a quintessential reservoir for obligate marine Lulworthiaceae fungi. Our current knowledge of the mycological diversity associated with seagrass in Hong Kong remains poor. We analyzed the diversity of fungi associated with the most widely distributed seagrass species in Hong Kong *Halophila ovalis* (Hydrocharitaceae), using a combination of culture-based methods and high-throughput amplicon sequencing. *Halophilomyces hongkongensis*, a novel fungal species in a newly proposed genus within the Lulworthiaceae family, was isolated from *H. ovalis* roots and rhizomes. The novel fungus showed distinct morphological characteristics, while both combined 18S-28S and internal transcribed spacer (ITS) phylogenetic trees based on maximum likelihood and Bayesian methods supported its discrimination from other existing Lulworthiaceae members. The ITS2 region in the Illumina sequencing results of multiple *H. ovalis* compartments, water, and adjacent non-seagrass sediments revealed continuous recruitment of *H. hongkongensis* by *H. ovalis* throughout the year despite dramatically fluctuating environmental conditions, with remarkably high proportions of this taxon found in root and rhizome internal tissues, possibly indicating a strong and specialized relationship established between the Lulworthiaceae fungal partner and its seagrass host. The inhibitory abilities exhibited by *H. hongkongensis* against *Staphylococcus aureus* SA29213 and ATCC 43300 (methicillin-resistant) may imply its capacity in producing (novel) antibacterial compounds. The discovery of *H. hongkongensis* as the first novel Lulworthiaceae taxon in Hong Kong, along with its distributional pattern in the seagrass meadow, provides valuable insights into the systematics and ecology of this strictly marine fungal family.

## 1. Introduction

The family Lulworthiaceae was first described, based on phylogenetic and morphological evidence, by Kohlmeyer et al. [1] as the sole family of the new order Lulworthiales, which only included the two genera *Lulworthia* and *Lindra* previously placed in the Halosphaeriales. Since then, another fourteen genera have been assigned to the Lulworthiaceae family, including *Cumulospora*, *Halazoon*, *Hydea*, *Haloguignardia*, *Kohlmeyeriella*, *Lulwoana*, *Lulwoidea*, *Matsusporium*, *Moleospora*, *Moromyces*, *Orbimyces*, *Rostrupiella*, *Sammeyersia* [2,3], and *Paralulworthia* [4]. The entire Lulworthiaceae family consists of obligate marine fungi collected worldwide from various substrates, such as seagrass, submerged/drifted wood, and seaweeds [5,6]. Among them, seagrass tissues represent one of the major sources for Lulworthiaceae fungi [7], which have been previously isolated from *Posidonia oceanica*, *Zostera marina*, *Thalassia testudinum*, *Cymodocea manatorum*, and other host seagrass plant species. The most recently described novel taxa in the Lulworthiaceae have been isolated in Italy from the Mediterranean seagrass *Posidonia oceanica* and ascribed to the novel genus *Paralulworthia* by Poli et al. [4,6].

Teleomorph–anamorph connection for Lulworthiaceae species has been rarely defined. Indeed, only a few such connections have been recognized among the approximately 55 species [2,3,8] of Lulworthiaceae fungi currently known [9,10]. For instance, *Lulwoana uniseptata* was originally linked to its anamorph *Zalerion maritima* through single ascospore cultivation [11]. Nakagiri and Tubaki [12] first connected *Anguillospora marina* (anamorph) with *Lindra obtusa* (teleomorph) through teleomorph induction from anamorphic culture. Marine-derived fungi are often in asexual morphs [2] and attempts to induce teleomorphs from marine anamorphic cultures are generally challenging and unsuccessful. Nutrient level, light, and substrates [13,14,15] are crucial factors for the induction of fungal resistant forms (ascomata, conidia, etc.).

The marine environment is expected to be a large reservoir for fungi, where only one-fifth of the more than 10,000 total estimated taxa have been described to date [2]. As one of the major components in marine food webs, fungi play an important role mainly as saprotrophs and symbionts [2,7]. Endophytic fungi are endobionts living within the host plant tissues asymptomatically, while sometimes acting as commensalistic, mutualistic, or weakly parasitic organisms based on a number of varying environmental conditions [7]. Seagrass, one of the most productive ecosystems on earth, has received relatively less attention concerning its associated fungi, whereas prokaryotic microorganisms inhabiting this ecosystem, especially bacteria, have been extensively analyzed [16]. Several studies investigated seagrass endophytic fungal diversity either through culture-based methods or high-throughput sequencing [6,17,18,19,20], but the ecological functions of seagrass endophytic fungi and the exact nature of seagrass–fungi interactions still remain unclear [21]. Seagrass endophytic fungi are known to be potential sources of antibacterial compounds. Previous studies have reported such compounds extracted from endophytic fungi of various seagrass species, such as *Cymodocea serrulata*, *Halophila ovalis*, *Thalassia hemprichii* [22,23], and *Zostera marina* [24]. In addition, some Lulworthiaceae members are lignocellulolytic enzyme producers and may therefore be involved in decomposing seagrass detritus [7].

In spite of the extensive distribution of seagrass meadows in Hong Kong’s marine environment, information on the seagrass-associated mycological diversity is scarce. To the best of our knowledge, only Alva et al. [25] have previously described the endophytic fungal assemblages in the Hong Kong seagrass *Zostera japonica* [25] and discovered that most of the analyzed fungal endophytes possess the ability to produce lignin-modifying enzymes. The present work intended to contribute to fill the gap in our knowledge on Hong Kong seagrass fungi. In particular, we investigated the associations between the most widely distributed seagrass species in Hong Kong [26], *Halophila ovalis* (Hydrocharitaceae), and fungi. *Halophila ovalis* endophytic fungi have previously been investigated in Thailand [27], India [28,29,30], and the Malay Peninsula [31]. However, in the latter study, no particular marine fungal linages were found and no detailed taxonomy of the *H. ovalis* associated fungi was provided.

The aim of this study is to assess the diversity of fungi that are inextricably associated with the seagrass *H. ovalis* using a combination of culture-based methods and high-throughput amplicon sequencing. Among the isolated fungi, we proposed a new marine genus, *Halophilomyces*, in the Lulworthiaceae, typified by the species *Halophilomyces hongkongensis*, which showed an absolutely dominant presence in *H. ovalis* roots and rhizomes. We analyzed the phylogenetic relationships of the described novel fungus within Lulworthiales and tested its antibacterial activity.

## 2. Materials and Methods

### 2.1. Sample Collection and Pretreatment

**Figure 1:**
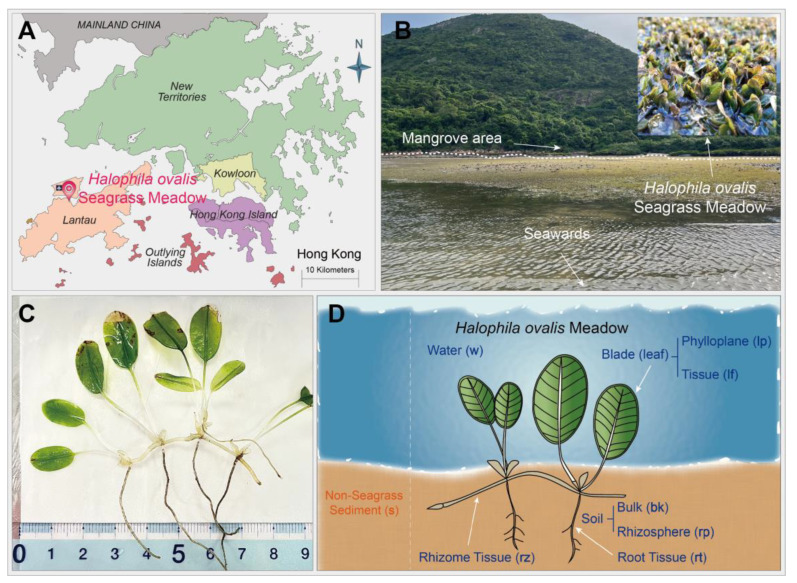
(**A**) The geographical location of the studied *Halophila ovalis* seagrass meadow in Hong Kong shown on a district map; (**B**) a view of the seagrass meadow and adjacent mangroves; (**C**) *H. ovalis* plant structure; (**D**) different sample types with their corresponding abbreviations for this study.

The studied *H. ovalis* seagrass meadow is located at an intertidal zone near San Tau Pier, in Tung Chung Bay, Lantau Island of Hong Kong (22°17′14″ N, 113°55′31″ E) (Figure 1A). Mangroves are distributed along the shore next to the seagrass meadow (Figure 1B), with *Aegiceras corniculatum* being the dominant mangrove species. The sampling was conducted in two distinct periods of the year characterized by dramatically different environmental conditions, specifically on 10 March 2023, during the dry season with cooler temperatures, and 24 August 2023, during the monsoon season associated with frequent/heavy rainfall, occasional tropical cyclones, higher humidity, and higher temperatures. The studied seagrass species *Halophila ovalis* is characterized by an extremely small size (Figure 1C). Samples were collected when the seagrass meadow was exposed to the air by the low tide. Three sampling plots (50 cm × 50 cm) were randomly selected in the studied seagrass meadow, each located 50 m apart from the others. Each plot included three subplots represented by the core. Seagrass leaves were first collected from each subplot and transferred to sterile plastic bags; then, the PVC pipes (10 cm in diameter) were inserted for core collection. Additionally, water samples above the seagrass canopy as well as bare sediments from an area near the seagrass meadow were collected (Figure 1D). In total, 18 seagrass cores and 18 leaf samples, together with 10 water and 6 non-seagrass sediment samples (Figure 1D), were obtained as replicates for Illumina sequencing. We collected an extra core during March sampling in an effort to isolate fungi from the roots, rhizomes, and rhizosphere soil of *H. ovalis*. All cores were transferred into autoclaved glass bottles and placed on ice with other samples in insulated containers for transport back to the laboratory. The collected samples were temporarily stored at 4 °C until the next treatment. Seagrass compartment separation and fungal isolation were conducted within 24 h of sample collection.

After a series of processing steps applied for core and leaf samples (described below), a total of 124 samples were subjected to total genomic DNA extraction. Seagrass roots, rhizomes, and rhizosphere and bulk soil (Figure 1D) from each core were separated using tools that were all well autoclaved, either flame- or alcohol-sterilized. First, the whole below-ground root and rhizome net were taken out from the glass bottle (meanwhile, the sediment from the corner of the core that contains nearly no seagrass tissues was treated as “bulk soil” and transferred to Eppendorf tubes, ready for downstream DNA extraction) and shaken vigorously to remove loosely attached sediment on the tissues. Subsequently, tissues with adhesive soil were transferred to 50 mL Falcon tubes containing an autoclaved phosphate-buffered saline (PBS) solution (0.01 M, pH 7.4), vortexed for 5 min [32], and then sonicated for 30 s at 50~60 Hz [33] to release the rhizosphere soil in the PBS solution. It was not possible to separate the roots and rhizomes before the above-mentioned washing and sonicating steps, because the wet sediments mixing the tiny structure of *H. ovalis* together made the different structures undistinguishable. Therefore, we treated the adhesive soil from the different below-ground tissues as a whole, under the name of “rhizosphere soil”. The cleaned seagrass tissues were picked from the liquid part containing the rhizosphere soil and placed on a Petri dish surface for root and rhizome separation using scissors, followed by surface sterilization to remove contaminants. The latter procedure consisted of the following steps: three cycles of vortex washing the roots or rhizomes using an autoclaved PBS solution until no visible soil particles were left, 1 min of soaking in 70% ethanol, and a final three cycles of rinsing with an autoclaved PBS solution before drying on a sterile gauze. The remaining “rhizosphere soil” solution contained in the 50 mL Falcon tubes was centrifuged at 6000× *g* for 15 min to pellet the soil. The supernatant was discarded, while the rhizosphere soil pellets, together with the surface-sterilized roots and rhizomes described above, were processed for fungal isolation and total genomic DNA extraction. Seagrass leaves were gently rinsed by autoclaved PBS to remove any attached materials on the surface. Then, the leaves were transferred to Falcon tubes and covered by autoclaved PBS, followed by vortexing, sonication, liquid–leaf separation, centrifugation, and surface sterilization of the leaves, as described above, to obtain the phylloplane and leaf tissue (Figure 1D) for total genomic DNA extraction.

### 2.2. Fungal Isolation and Molecular Identification

Fungal isolation from fresh *H. ovalis* root and rhizome fragments and rhizosphere soil was performed shortly after sampling. Surface-sterilized roots and rhizomes were cut into 5 mm segments and placed on Potato Dextrose Agar (PDA) medium, amended with 100 mg/L penicillin and streptomycin to prevent bacterial contamination. A total of nine root and rhizome segments were placed on PDA. The effectiveness of surface sterilization was checked by pressing a set of root and rhizome portions on PDA media. For the rhizosphere soil, 10- and 100-times diluted suspensions were prepared from well-grounded soils, and then, 100 μL of uniform soil suspensions from each dilution were placed on PDA with antibiotics. All plates were sealed, incubated in darkness at 25 °C, and checked daily. In the plates for fungal isolation from rhizosphere soil, the colonies started to appear after 1 to 2 days of incubation, while the mycelia from the plates for endophytic fungal isolation started to grow after 7 days of incubation. Newly developed fungal colonies were picked and transferred to new PDA media to obtain pure fungal strains. Fungal mycelia from pure cultures were scraped and DNA was extracted using the DNeasy PowerSoil Pro Kit (QIAGEN, Hilden, Germany) according to the manufacturer’s protocol. Primers ITS1 and ITS4 were used for PCR amplification of the fungal internal transcribed spacer (ITS) region. For fungal strains of particular interest for their potential specific association with *H. ovalis* (Lulworthiaceae isolates), small subunit (SSU) and large subunit (LSU) rDNA were also amplified [34] using NS1, NS2, NS3, and NS4 for SSU, and LR0R, LR3, LR5, and LR7 for LSU. PCR amplifications were performed following Wang and Pecoraro [35], except for the modification of the extension step for SSUs and LSUs to 72 °C for 30 s instead of 15 s. PCR products were Sanger-sequenced at BGI Genomics (Hong Kong). The SSU and LSU sequences were aligned and assembled using Geneious Prime (version 2022.2.2). All sequences were BLASTn-searched in NCBI to determine the closest matching sequences and deposited in GenBank under accession numbers PP347844–PP347853 (SSU sequences), PP347858–PP347867 (LSU sequences), and PP350734–PP350759 (ITS sequences).

### 2.3. Morphological Characterization and Teleomorph Induction

Fungal morphology was characterized based on macroscopic and microscopic observations [36,37]. Microscopic features of fungal isolates were observed under a Nikon ECLIPSE Ni-U microscope (Nikon, Tokyo, Japan) and a Leica M205 FCA stereo microscope (Leica Microsystems, Wetzlar, Germany). For some strains, the slide culture method [14] was used for observations in order to obtain a better visual of important structures (e.g., conidial ontogeny and conidiophore arrangements).

We attempted to stimulate sexual reproduction of *H. ovalis* endophytic Lulworthiaceae strains by applying various methods, including fungal cultivation on different natural substrates, slide culture, incubation under 12 h of light followed by 12 h of darkness, cold shock, damaging of the colonies by scratching, etc. [13,14,38,39]. For the cultivation of Lulworthiaceae isolates on natural substrates, double-autoclaved plant materials from a variety of sources, including the host seagrass, wood pieces (from roots and branches) and leaves of the nearby mangrove species *A. corniculatum*, and banana leaves, were inoculated. PDA medium cubes (1 cm^3^) containing fungal hyphae were cut from the outer edge of actively growing Lulworthiaceae strains (3 weeks old) and inoculated onto two sets for each of the above-described natural substrates placed inside Petri dishes. The plates were sealed with parafilm and incubated at 25 °C, with one set in darkness and another under a light/dark cycle (12/12 h). The above-mentioned incubation conditions were also applied for slide cultures and Petri dish PDA cultures. Fungal growth was observed continuously by naked eyes and microscopes until the media dried out or became contaminated.

### 2.4. Phylogenetic Analysis

Phylogenetic trees based on maximum likelihood (ML) and Bayesian Inference (BI) analyses of combined SSU-LSU rDNA and ITS datasets were produced to understand the relationships between *H. ovalis*-associated Lulworthiaceae fungi and selected database sequences. In particular, the reference nucleotide sequences of SSU and LSU rDNA were selected from phylogenetic studies focusing on the order Lulworthiales performed by Campbell et al. [9] and Poli et al. [6]. These sequences mainly included diverse Lulworthiales members from multiple marine sources, including seagrasses, seawater, driftwoods, submerged woods, etc., together with representatives of the orders Xylariales, Sordariales, Phyllachorales, Halosphaeriales, Microascales, Hypocreales, and Pleosporales, which were closely related to the taxonomic history of Lulworthiales [1]. Additionally, SSU and LSU rDNA sequences of the top two best matches (Lulworthiaceae sp. strain SLF 0120.1411, Lulworthiaceae sp. strain SLF 0117.0203) of our Lulworthiaceae isolates’ LSU sequences from the BLASTn search were included in the corresponding dataset. For the ITS phylogenetic tree, Lulworthiales sequences mainly from seagrasses [6,20] and other substrates [40], together with the best BLASTn match sequence, were included in the dataset. The trees were rooted with Pleosporales species following previous studies [6,40].

Multiple sequence alignment was conducted using MUSCLE (version 3.8.31) with the default setting designed for providing the best accuracy [41] and visually inspected using AliView v.1.28. Alignment trimming was performed using trimAI (version 1.4.1) with “-automated1”, which was optimized for maximum likelihood tree construction, relying on the heuristic selection of an automated statistical method (strict, strictplus, or gappyout) for trimming [42]. For ML analyses, the best fit models (TIM2+F+G4 for the SSU-LSU tree TIM2e+I+G4 for ITS tree) were selected using ProtTest v.3.4.2 [43]; then, phylogenetic trees were constructed using IQ-TREE v.1.6.12 [44] with the ultrafast bootstrap parameter “-bb 1000” [45]. BI analyses were performed in MrBayes 3.2.7 [46] with the GTR evolutionary model (lset nst = 6 rates = invgamma). Four Markov Chain Monte Carlo (MCMC) simulations were set to run alignments for 10 million generations (ngen = 10,000,000), sampling every 100 generations (samplefreq = 100). The trees from the first phase were discarded (burninfrac = 0.25), and the remaining were used for a majority rule consensus tree (sumt) with posterior probabilities. The trees were finally visualized using Interactive Tree Of Life iTOL v.6 [47]. The topological structure of trees based on ML and BI methods were similar; therefore, ML trees with bootstrap values (BS) and Bayesian posterior probabilities (PPs) are ultimately presented.

### 2.5. Preliminary Antibacterial Assay

The antibacterial activity test of fungi isolated from the *H. ovalis* seagrass meadow was performed against various pathogenic microorganisms, including the Gram-positive bacteria *Staphylococcus aureus* SA29213, the methicillin-resistant *S. aureus* (MRSA) ATCC 43300, the Gram-negative bacteria *Escherichia coli* ATCC 25922, and high-virulence *Klebsiella pneumoniae* Hvkp2. The agar plug diffusion method was applied for the antibacterial screening using fungal strains cultured on PDA at 25 °C for 10 days, except in the case of Lulworthiaceae isolates where 21-day-old cultures were used due to their slow growth. Briefly, overnight incubated pathogenic bacteria were transferred to fresh Luria–Bertani (LB) broth with the ratio 1:100 and then cultured at 37 °C and 250 rpm till the optical density (OD) reached 0.5. The bacterial cultures were centrifuged at 6500 rpm for 5 min. The supernatant was discarded, and the bacterial pellets were resuspended in 0.85% saline. The above-mentioned washing procedure was repeated twice. Then, the amount of bacteria was adjusted to approximately 5 × 10^6^ CFU/mL. One-hundred microliters of bacterial suspensions were spread on LB medium plates and air-dried. Triplicate plugs (7 mm in diameter) of each fungal strain were transferred to the LB plates containing each test bacterium. The inoculated plates were kept at 4 °C for 19 h to allow for the diffusion of metabolites and then cultured at 37 °C overnight to enable the growth of test bacteria. The inhibition zones were measured after incubation. Colistin, tigecycline, and vancomycin (4 mg/mL) were used as the positive control for *E. coli*, *K. pneumoniae,* and *S. aureus*, respectively.

### 2.6. Fungal ITS2 Illumina Sequencing and Data Processing

Total genomic DNA of 124 samples were extracted using the DNeasy PowerSoil Pro Kit (QIAGEN), the DNeasy PowerBiofilm Kit (QIAGEN), and the DNeasy Plant Pro Kit (QIAGEN). We used nested PCR [48,49] to increase the specificity and sensitivity of PCR amplification on seagrass fungi. The first round of PCR was performed with the primer pair ITS1F-ITS4 to target the whole fungal ITS region. The products of the first-round PCR were used as templates for the second-round PCR amplification of the fungal ITS2 region, using the primers fITS7 (5′-GTGARTCATCGAATCTTTG-3′) and ITS4 (5′-TCCTCCGCTTATTGATATGC-3′) linked with barcodes. PCR reactions were carried out in 25 μL solutions composed of 12.5 μL 2 × Phusion^®^ High-Fidelity PCR Master Mix with HF Buffer (New England Biolabs, MA, USA), 1.25 μL 10 mM of each primer, and 10 ng template DNA. The PCR reaction conditions were set as follows: initial denaturation at 98 °C for 30 s, 32 cycles of denaturation at 98 °C for 10 s, annealing at 54 °C for 30 s and extension at 72 °C for 45 s, and final extension at 72 °C for 5 min. For the second-round PCR, the amplifications were prepared in duplicates (25 μL × 2). An amplicon library was prepared by adding index codes to the PCR products following the instructions on Illumina’s amplicon library preparation guide. Equimolar amounts of amplicons were pooled and pair-end sequenced using the NovaSeq 6000 PE250 platform (Illumina Inc., San Diego, CA, USA). Adapters and barcodes from the raw data were removed subsequently. The raw sequencing fastq files were deposited in the Sequence Read Archive (SRA) at the National Center for Biotechnology Information (NCBI) as BioProject ID PRJNA1062533.

Fungal ITS2 sequencing data were processed using QIIME 2 pipeline (version 2023.2.0). Briefly, raw reads were imported and demultiplexed. Read quality was visually inspected, and then, DATA2 was used to filter low-quality reads, denoise sequences, merge paired reads, and eliminate chimeras. The chimera-free and quality-filtered sequences were clustered into operational taxonomic units (OTUs) at a 97% similarity level using the “cluster-features-de-novo” command via the qiime vsearch plugin. The taxonomic profiles of OTUs were assigned using the “feature-classifier classify-sklearn” command with a confidence score of 0.9 (--p-confidence 0.9), referring to the pretrained classifier based on UNITE Database v9.0. Taxonomic information, abundances, and representative sequences of OTUs were extracted for further analyses from the files “taxonomy.qza”, “table.qza”, and “rep-seqs.qza”, respectively.

Fungal community diversity was analyzed and visualized using the R (version 4.2.2) packages phyloseq (v 1.46.0), tidyverse (v 2.0.0), ape (v 5.7-1), ggpubr (v 0.6.0), and microViz (v 0.11.0). Relative abundances of different fungal genera were calculated and presented by bar plots to visualize the distribution of the dominant genus (“*Lulwoana*”) in multiple samples/sample types. Wilcoxon rank-sum tests were used to explore variations in the relative abundance of “*Lulwoana*” among different sample types. Moreover, the representative sequence of “*Lulwoana*” detected by Illumina sequencing was added to the ITS phylogenetic tree to elucidate its relationship with Lulworthiaceae isolates from *H. ovalis*.

## 3. Results

### 3.1. Fungal Isolates and Microscopy

Ten fungal strains were isolated from *H. ovalis* plant tissues, mostly from rhizomes, except HOMAR 9 and HOMAR 10 that were isolated from roots (Table 1). All of the ITS, SSU, and LSU sequences of these strains shared similar best BLASTn hits (Table 1). *Lulwoana uniseptata*, in the Lulworthiaceae family, was found to be the best match for both ITS and SSU sequences, with identity near 100%. The LSU sequences of these ten strains matched with Lulworthiaceae sp. from an unpublished study related to alkalitolerant Lulworthiaceae fungi on the coasts of saline inland lakes, with identity ranging from 98.63% to 98.77%.

A total of sixteen fungal strains belonging to the five genera *Penicillium* (five species, seven strains), *Aspergillus* (four species, four strains), *Trichoderma* (one species, three strains), *Coprinellus* (one species, one strain), and *Nectria* (one species, one strain) were isolated from rhizosphere soil samples (Table S1). The colony and microscopic features of these five genera are reported in Figure S1.

### 3.2. Phylogeny

**Figure 2:**
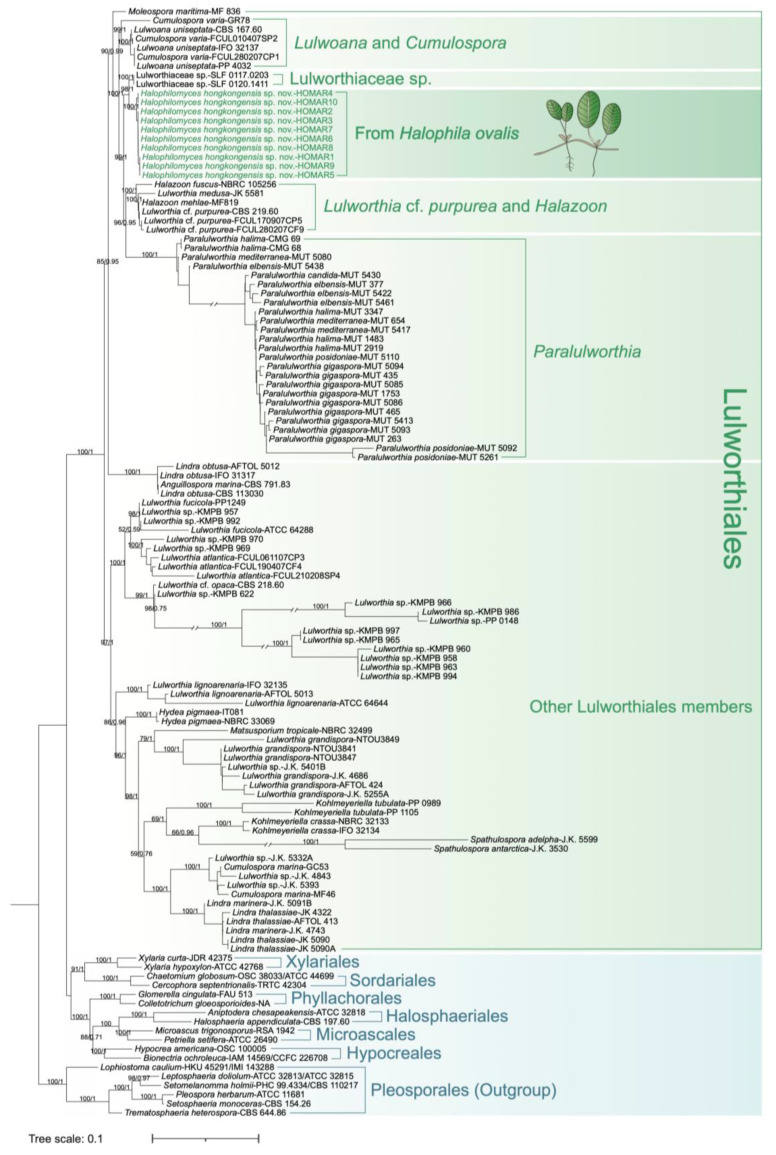
A phylogenetic tree derived from maximum likelihood analysis based on the combined SSU and LSU dataset constructed in IQ-TREE, with 1000 ultrafast bootstraps using the TIM2+F+G4 model selected by ProtTest. Bootstrap (BS) values (>50) from the maximum likelihood analysis followed by posterior probability (PP) values (>0.50) from the Bayesian analysis are added to the left of each node (BS/PP). The tree is rooted on the Pleosporales species. The symbol (//) on the branches refers to branch lengths presented at 1/2 of the actual length.

A total of 122 sequences (including *H. ovalis* strains), representing a large variety of Lulworthiales taxa isolated from numerous environmental sources, were included in the SSU-LSU phylogenetic tree (Figure 2). These Lulworthiales sequences were segregated into two robust groups (BS = 100%, PP = 1). The ten *H. ovalis* strains isolated in our study from seagrass root/rhizome clustered into a single separated clade “From *Halophila ovalis*” (Figure 2), which was a sister clade (BS = 98%, PP = 1) of “Lulworthiaceae sp.” represented by the two LSU sequence best matches from the BLAST search (SLF 0120.1411 and SLF 0117.0203; Table 1). The above-mentioned two clades were part of a bigger cluster with strong bootstrap and posterior probability support (BS = 100%, PP = 1), including “*Lulworthia* cf. *purpurea* and *Halazoon*” and “*Paralulworthia*” sister clades (BS = 99%, PP = 1), and the clade “*Lulwoana* and *Cumulospora*”, which contained the HOMAR SSU best match from the BLASTn search (*Lulwoana uniseptate* strain CBS 167.60; Figure 2).

**Figure 3:**
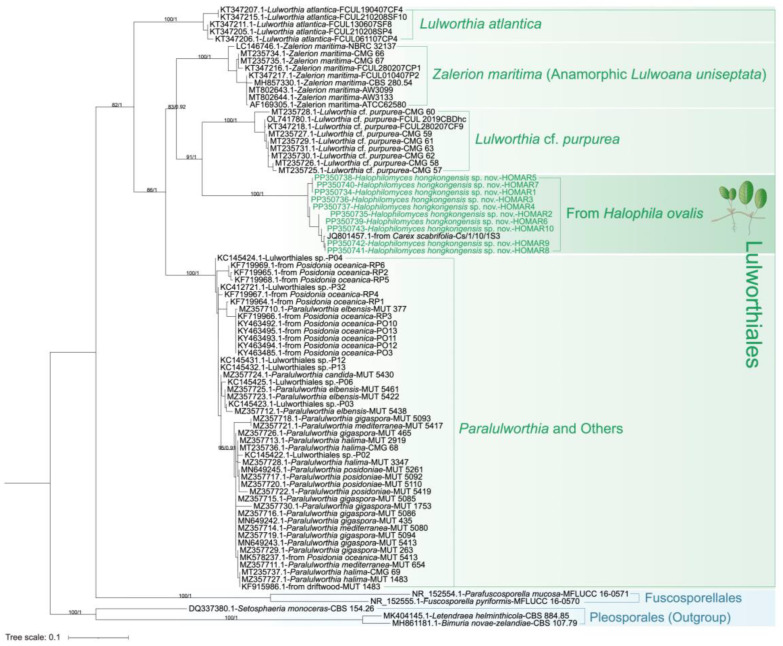
A phylogenetic tree derived from maximum likelihood analysis based on the ITS dataset constructed in IQ-TREE, with 1000 ultrafast bootstraps using the TIM2e+I+G4 model selected by ProtTest. Bootstrap (BS) values (>50) from the maximum likelihood analysis followed by posterior probability (PP) values (>0.50) from the Bayesian analysis are added to the left of each node (BS/PP). The tree is rooted on the Pleosporales species.

The ML tree of the ITS region contained 85 sequences (Figure 3). Consistent with the SSU-LSU tree, the endophytic fungi from *H. ovalis* formed an independent clade. This clade also included the sequence of their best match, which was previously obtained by Seo et al. 2012 [50] from a fungus described in NCBI as “*Lulwoana uniseptata*” (Table 1), isolated from the halophytic plant *Carex scabrifolia* in Suncheon Bay, South Korea (Figure 3). The *H. ovalis* isolates were phylogenetically close to the fungi included in the sister clade “*Lulworthia* cf. *purpurea*”.

Based on both SSU-LSU and ITS trees, we proposed to include the endophytic fungi isolated from *H. ovalis* into the novel genus *Halophilomyces*, typified by the new species *Halophilomyces hongkongensis*.

### 3.3. Taxonomy

***Halophilomyces*** X. Wang, L. Pecoraro & H.B. Liu, gen. nov.

MycoBank ID: MB854689

*Type species*: *Halophilomyces hongkongensis* sp. nov.

*Etymology*: Referring to the host seagrass species *Halophila ovalis*.

*Diagnosis*: Hyphae with abundant loop structures. Chlamydospores in chains. Conidia generally increasing in size from base to apex. Single-celled conidia present. Colonizing seagrass inner tissues.

***Halophilomyces hongkongensis*** X. Wang, L. Pecoraro & H.B. Liu, sp. nov.

MycoBank ID: MB854690

*Type*: Hong Kong SAR, South China Sea, Lantau Island, intertidal zone, 22°17′14″ N, 113°55′31″ E, from seagrass *Halophila ovalis* rhizomes, 10 March 2023, leg. X. Wang. Ex-type living culture (HOMAR2) preserved in a metabolically inactive state at LP Culture Collection (personal culture collection held in the laboratory of Prof. Lorenzo Pecoraro) at the School of Pharmaceutical Science and Technology, Tianjin University, Tianjin, China, and at the Hong University of Science of Technology, Hong Kong SAR.

*Additional examined material*: Hong Kong SAR, South China Sea, Lantau Island, intertidal zone, 22°17′14″ N, 113°55′31″ E, from seagrass *Halophila ovalis* roots, 10 March 2023, leg. X. Wang, HOMAR9.

*Etymology*: Referring to the location where the fungus was collected.

*Diagnosis*: *Halophilomyces hongkongensis* differs from its close phylogenetic neighbors *Lulworthia* cf. *purpurea*, *Halazoon,* and *Paralulworthia* in the conidia which gradually increase in size, the presence of abundant loop-structured hyphae, and the host (seagrass *Halophila ovalis*).

**Figure 4:**
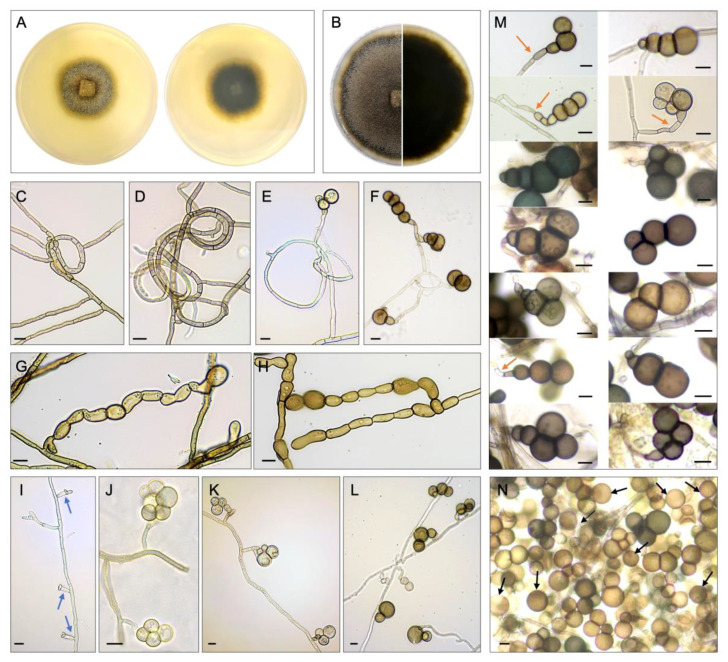
*Halophilomyces hongkongensis* sp. nov. colony and microscopic features. Fourteen-day (**A**) and thirty-day (**B**) colony morphology at 25 °C on PDA incubated under light/dark cycle (12/12 h) and reverse view; (**C**,**D**) loop structures formed by fusion of hyphae; (**E**,**F**) young and mature conidia arising from loop structures; (**G**,**H**) multicellular chlamydospores; (**I**) conidiogenous cells (indicated by blue arrows); (**J**,**K**) young conidia and conidiophores; (**L**) mature conidia and conidiophores; (**M**,**N**) conidia. Orange arrows indicate conidiophores bearing conidia. Black arrows indicate single-celled conidia. Scale bar = 10 μm.

*Description*: Hyphae 1.9–5.3 μm wide, septate, hyaline, olivaceous or brown, abundant loop structures (Figure 4C–F). Chlamydospores in chains, cells ellipsoidal, brown or olivaceous (Figure 4G,H). Conidia pleurogenous, solitary, coiled, 24.5–53.8 μm in diameter, 1–3 (4) septa, constricted at the septa, dark olivaceous or brown; conidial cells globose to subglobose, generally increasing in size from base to apex (Figure 4I–N): apical cell 14.1–24.8 μm, basal cell 9.1–21.5 μm. Single-celled conidia (9.5–29 μm) also common (Figure 4N). Teleomorph not observed.

*Colony description*: Colonies cultured on PDA reaching 30–35 mm and 35–43 mm at 25 °C under light/dark cycle (12/12 h) and darkness (14 days), respectively. Mycelium partly superficial, partly immersed, effuse, at first olivaceous, lighter at the edges (Figure 4A), becoming darker and brownish when old (Figure 4B); reverse dark olivaceous color with lighter edges (Figure 4A), becoming darker when old (Figure 4B).

*Distribution and habitat*: Hong Kong SAR. Actively colonizing root and rhizome internal tissues of the host seagrass *Halophila ovalis*.

*Notes*: The genus *Halophilomyces* formed a highly supported monophyletic clade phylogenetically close to *Lulworthia* cf. *purpurea*, *Halazoon*, *Paralulworthia,* and Lulworthiaceae isolates (LSU best BLASTn hits) in the combined SSU-LSU tree, while it was a sister clade of *Lulworthia* cf. *purpurea* in the ITS tree. The conidial structures of *H. hongkongensis* shared certain similarities, represented by the multi-septate and irregularly coiled conidia, with *Lulwoana uniseptata*, which was the best BLASTn hit of *H. hongkongensis* ITS and SSU sequences. The increasing diameter from base to apex of *H. hongkongensis* conidia represents a distinct morphological characteristic compare to other Lulworthiales members.

### 3.4. Teleomorph Induction of Halophilomyces hongkongensis

**Figure 5:**
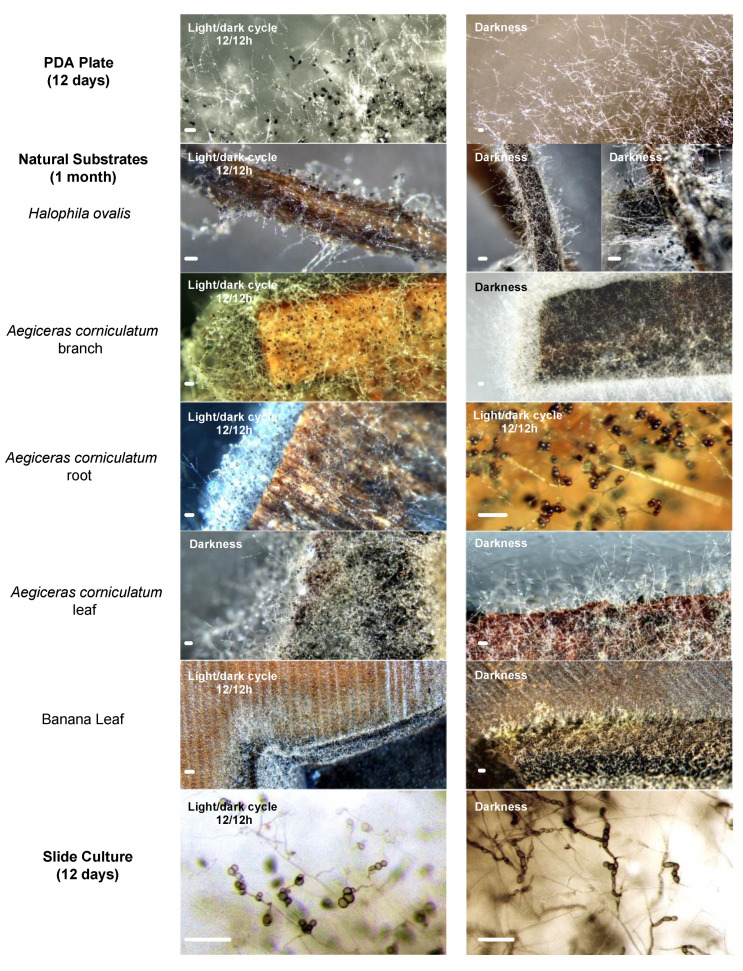
*Halophilomyces hongkongensis* at 25 °C cultured under different environmental stimuli on PDA (figure shows growth edge of 12-day-old colonies), host seagrass tissues and other natural substrates (1-month-old), and slide cultures (12-days-old). Features observed by stereo microscope. Light/dark cycle (12/12 h) and darkness in figure indicate incubation conditions. Scale bar = 100 μm.

During the process of teleomorph induction from anamorphic cultures, no sexual reproductive structure of *H. hongkongensis* was observed after culturing colonies on natural substrates, glass slides, under a 12/12 h light/dark cycle, etc. Nonetheless, we found some of these approaches useful to induce the asexual sporulation of the studied fungal species. Mycelia barely extended on banana leaf disks either under a 12/12 h light/dark cycle or complete darkness during incubation (Figure 5). The spore production of *H. hongkongensis* was apparently enhanced by the introduction of light, which resulted in the formation of the dark-colored conidia extensively covering all culture substrates, including PDA, host seagrass tissues, *A. corniculatum* branch and root, and slide cultures, with the sole exception of banana leaves (Figure 5). Marine-linked substrates (seagrass and mangrove tree tissues) were suitable for inducing sporulation of *H. hongkongensis* even under darkness, although occasionally, the conidia were not present in certain parts of the seagrass and mangrove leaf cultures. The slide culture method did not show significant effects on conidia induction of the analyzed strains, while chlamydospores were mostly produced from cultures under darkness.

### 3.5. Antibacterial Assay

**Figure 6:**
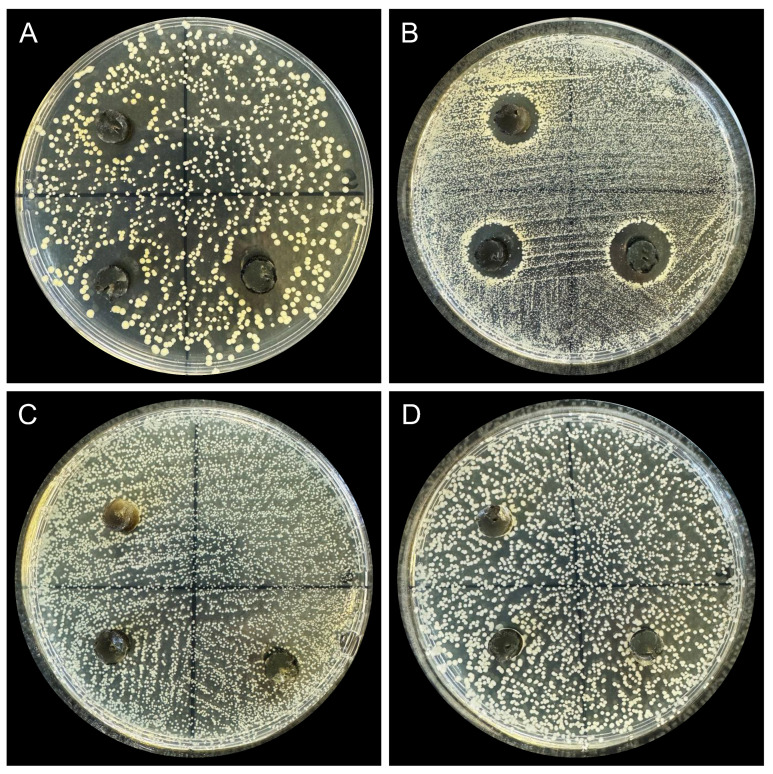
Agar plug diffusion assay of *Halophilomyces hongkongensis* against four pathogenic bacteria: (**A**) *Staphylococcus aureus* SA29213, (**B**) *S. aureus* (MRSA) ATCC 43300, (**C**) *Escherichia coli* ATCC 25922, and (**D**) *Klebsiella pneumoniae* Hvkp2.

The novel fungal species *Halophilomyces hongkongensis* isolated from the seagrass *H. ovalis* showed clear antimicrobial activity by producing inhibition zones against *S. aureus* SA29213 (Figure 6A) and *S. aureus* (MRSA) ATCC 43300 (Figure 6B) with a diameter ranging from 10.5 to 16.33 mm and from 11.5 to 14.67 mm, respectively. On the contrary, no inhibition zone was observed for Gram-negative bacteria tests (Figure 6C,D). For the other fungal strains collected from *H. ovalis* rhizosphere soil tested by agar plug diffusion assay, *Aspergillus westerdijkiae* was the sole fungus that inhibited all four test bacteria (Table S2), while *Nectria* sp. produced the largest inhibition zones against *S. aureus*.

### 3.6. Fungal Community Structure

A total of 22,011,744 raw reads and 15,784,425 effective sequences were generated from fungal ITS2 Illumina sequencing. The sequences from each sample ranged from 57,473 to 300,371. After the removal of non-fungal sequences, 2097 to 16,944 sequences remained. We normalized the OTU table by resampling 2097 sequences per sample. Among the 1243 OTUs assigned to the fungal kingdom (Tables S3 and S4), 846 were assigned to Ascomycota, 134 to Basidiomycota, 78 to Rozellomycota, 10 to Chytridiomycota, and 5 to Aphelidiomycota, whereas 170 OTUs remained unassigned at the phylum level.

**Figure 7:**
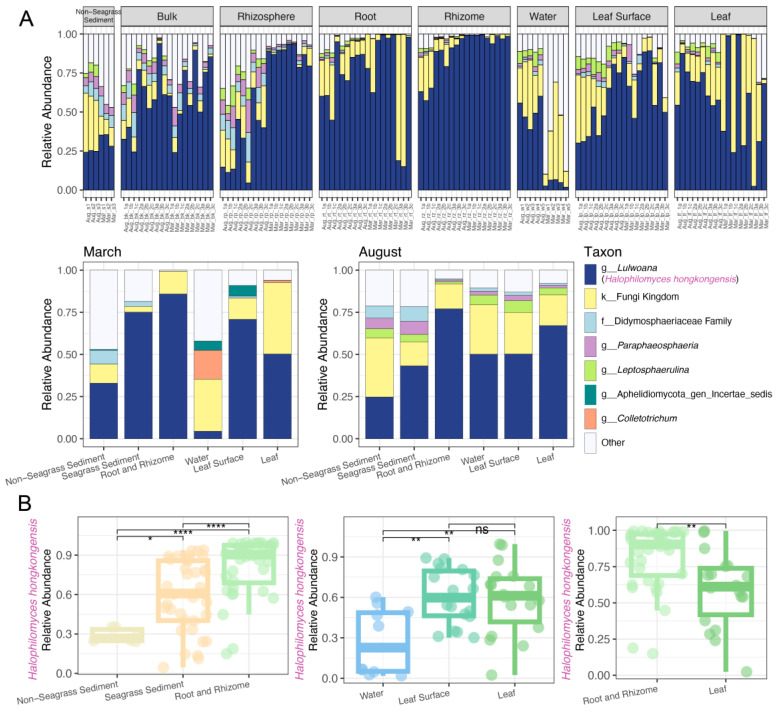
The relative abundance of fungi. (**A**) The genus-level relative abundance of the top 5 fungal taxa for each sample and sampling month (March and August). (**B**) Comparisons of *Halophilomyces hongkongensis* relative abundance in different sample types. Differences between groups evaluated by Wilcoxon rank-sum test are indicated as follows: * *p* < 0.05, ** *p* < 0.01, **** *p* < 0.0001, and ns = not significant. In this figure, the original genus “*Lulwoana*” classified according to UNITE Database v9.0 was changed to the novel species *Halophilomyces hongkongensis* based on phylogenetic evidence, which showed clustering of the “*Lulwoana*” representative OTU with *Halophilomyces* isolates in an independent clade. The sample ID consists of the sampling month (MAR or AUG), sample type (s = non-seagrass sediment, bk = bulk soil, rp = rhizosphere soil, rt = root, rz = rhizome, w = water, lp = leaf phylloplane, and lf = leaf), and replicate information. Seagrass sediment under sample type is composed of both bulk and rhizosphere soil from the seagrass meadow. In the taxa names, “k” = kingdom, “f” = family, and “g” = genus.

At the genus level, the relative abundance of “*Lulwoana*”, which only included one species *Lulwoana uniseptata*, was always found to be the highest across different sampling periods (i.e., March and August; Figure 7A, Table S5). Twelve OTUs were classified as “*Lulwoana*” at the genus level, but we only treated one OTU (ID: 1061269a449f32ed85148edd5cefe57e) as the representative of the “*Lulwoana*” community, which included 160,513 out of 160,535 “*Lulwoana*” sequences detected. Other “*Lulwoana*” OTUs were considered rare because they were detected only a few times (Table S5). Therefore, we added the sequence of this representative OTU to the ITS phylogenetic tree (Figure 3) to elucidate the relationship between “*Lulwoana*” obtained from the culture-independent method and the *Halophilomyces hongkongensis* isolates whose ITS sequences also primarily matched with *Lulwoana uniseptata* based on the BLASTn search (Table 1). The phylogenetic tree based on ML and BI methods showed that the “*Lulwoana*” representative OTU detected by Illumina sequencing clustered with *H. hongkongensis* isolates in a strongly supported (BS = 100%, PP = 1) independent clade (Figure S2), which confirmed that “*Lulwoana*” actually corresponded to the novel fungal taxon. Therefore, we replaced the name “*Lulwoana*” with *H. hongkongensis* in the Illumina sequencing results (Figure 7).

Among multiple sample types, the relative abundance of *H. hongkongensis* increased significantly from non-seagrass sediments to seagrass sediments, seagrass roots, and rhizomes (Figure 7B). A higher relative abundance of *H. hongkongensis* was found in leaf internal tissues and on the leaf surface compared to water sampled above the seagrass canopy (Figure 7B). *Halophila ovalis* below-ground plant tissues (roots and rhizomes) harbored significantly higher proportions of the novel fungal taxon compared to the leaf tissues (Figure 7B).

## 4. Discussions

This study represents an important contribution to the knowledge of the diversity and ecology of seagrass-associated fungi in Hong Kong’s marine environment. The isolation of Lulworthiaceae mycobionts colonizing the internal root and rhizome tissues of the seagrass species *Halophila ovalis* enabled the identification of the novel fungal taxon *Halophilomyces hongkongensis* using multi-locus phylogenetic analyses and morphological observations, while the combined ITS high-throughput sequencing approach allowed for the screening of *H. hongkongensis* presence in different tissue and sediment types from a *H. ovalis* meadow. The combined results from the different applied analytical methods add value to the discovery of the novel Lulworthiaceae fungi, not only from a taxonomic point of view [6,40] but also in terms of understanding the potential ecological roles that the newly described seagrass-associated fungi may play in the plant partnership and in the whole seagrass meadow environment.

Very few novel Lulworthiales taxa were reported in recent years, including *Lulworthia atlantica* [40], *Paralulworthia* [4,6], and *Paramoleospora* [51]. Fungal species in the Lulworthiaceae family have been rarely recorded in Hong Kong, mostly isolated from inter-tidal mangrove woods, including *Lulwoana uniseptata*, *Sammeyersia grandispora* (=*Lulworthia grandispora*), *Hydea pygmea*, *Halazoon fuscus,* and *Cumulospora marina* [52]. Our findings of *H. hongkongensis* provide the first novel Lulworthiaceae species discovered in Hong Kong and add a new record of seagrass host (i.e., *H. ovalis*) for this fungal family, thus increasing our knowledge on the ecological interactions between Lulworthiaceae fungi and marine plants.

The ability of *H. hongkongensis* to fight against pathogenic Gram-positive Methicillin-resistant *S. aureus* may indicate its potential in producing antibacterial compounds. Jenssen et al. [53] isolated the first novel bioactive secondary metabolite of Lulworthiales from a Lulworthiaceae fungus (strain 067bN1.2) obtained from driftwood in Norway, named Lulworthinone, with antibacterial activity against clinical methicillin-resistant *S. aureus*. Interestingly, the Lulworthiaceae fungus from the study of Jenssen et al. [53] was placed into a cluster with “*Paralulworthia*” and “*Lulworthia* cf. *purpurea* and *Halazoon*” (sister clade) in the combined 5.8S-SSU-LSU phylogenetic tree, similar to the placement of our *H. hongkongensis* which also showed a closer relationship with the above-mentioned two groups. It is probably more than a coincidence that our *H. hongkongensis* exhibited anti-MRSA capability. Further efforts are required to extract and identify the natural compounds from *H. hongkongensis* to confirm its capacity for antimicrobial metabolite production and biotechnological applications.

High-throughput sequencing detected a group of OTUs that were originally classified as “*Lulwoana uniseptata*” according to UNITE Database. Considering the fact that the ten isolated endophytic fungal strains from the root and rhizome segments also shared the same identity with these OTUs, according to the ITS sequence BLASTn search, we speculated that the “*Lulwoana uniseptata*” group found from high-throughput sequencing is actually identical to the novel taxon *H. hongkongensis*. In fact, we noticed that only one “*Lulwoana*” OTU was contributing to the abundance of this group in the fungal community (Table S5), and this OTU showed the same best BLASTn match (*Lulwoana uniseptata* strain Cs/1/10/1S3, JQ801457.1), which fell within the same strongly supported single clade as our isolated *Halophilomyces* strains (Figure S2). Therefore, we were able to use the data from metabarcoding analyses in order to define the relative abundance of *H. hongkongensis* in various sample types, thus providing a complete picture of the distribution of the novel fungus in the studied seagrass ecosystem. *Halophilomyces hongkongensis* dominated *H. ovalis* tissues, even across different periods of the year characterized by significantly different environmental conditions, showing a stable and persistent colonization of the seagrass by the associated fungus, which might suggest the establishment of a strong relationship between the two partners. The increasing abundance of *H. hongkongensis* from non-seagrass sediments to seagrass sediments, below-ground seagrass roots, and rhizomes, and from seagrass-surrounding water to leaves (surface), indicates specialized recruitment of *H. hongkongensis* in *H. ovalis* seagrass meadows. In terrestrial environments, fungi are often involved in mutualistic relationships with the host plants by forming mycorrhizal structures within the plant root system, which allow the plant to obtain additional nutrients, mainly inorganic, and water [54]. Seagrass living in marine environments is able to absorb nutrients from interstitial water through its roots and horizontal stems (rhizomes) buried in the sediment and from ambient water columns through the leaves [55]. Therefore, seagrass does not necessarily need the help of mycorrhizal fungi for nutrient/water uptake. Indeed, the four seagrass families (Zosteraceae, Hydrocharitaceae, Posidoniaceae, and Cymodoceaceae) are regarded as non-mycorrhizal [56]. The presence of the novel fungal taxon in the roots and rhizomes of the studied seagrass still does not allow us to make conclusions about the either beneficial, neutral, or detrimental effects of *H. hongkongensis* on its *H. ovalis* host. Further physiological studies are needed to understand whether or not a trophic relationship exists between the two associated organisms and to clarify the potential role that *H. ovalis* fungal partners may play in the carbon cycling process of seagrass meadows. Correlating the *Halophilomyces* community with environmental nutritional parameters, tissue section microscopy, manipulative inoculation experiments, and multi-omics approaches will help to disentangle the exact ecological function of the endophytic fungus *H. hongkongensis* in the seagrass meadow ecosystem.

The “*Lulwoana uniseptata*” isolated from the root of *C. scabrifolia* in a previous study by Seo et al. [50] clustered with *H. hongkongensis* in the ITS phylogenetic tree, which demonstrated the inaccurate identification proposed in the aforementioned work [50] based only on ITS sequence BLAST searches. In addition, the discovery of Lulworthiales fungi from the roots of the terrestrial halophyte *C. scabrifolia* by Seo et al. [50] is surprising since it is in contrast with the previously known ecology of Lulworthiales, considered a strictly marine fungal order. However, the *C. scabrifolia* individuals that Seo et al. [50] sampled were actually distributed in the coastal area of Suncheon bay in South Korea that receives splashing seawater regularly; this may have been the source of Lulworthiales fungi in *C. scabrifolia*.

Our numerous attempts to trigger sexual reproduction in the isolated strains of *H. hongkongensis* were unsuccessful, in spite of the multiple stimuli applied. As Poli et al. [6] summarized from the analysis of previously published studies, strictly vegetative growth is a common feature found in a large number of marine fungi. Culture conditions are crucial in laboratory experiments to stimulate marine fungi reproduction due to the adaptation of such fungi to the special high salinity and high hydrostatic pressure of the marine environment. The supplementation of sterile seawater in the culture media, either solid or liquid [57], which provides necessary salts with other marine components to better mimic the natural habitat and the exposure to near-UV light [58], may benefit teleomorph induction. More importantly, it should be noted that the sex of ascomycetous fungi is determined by a specific region in the fungal genome called the mating-type locus (MAT) [59]. Sexual reproduction of heterothallic fungi (self-sterile) requires mating with a strain with the opposite mating type [60], while homothallic fungi (self-compatible) can reproduce sexually within a single organism because the same strain and nucleus of these fungi contain both idiomorphs (MAT1-1 and MAT1-2) or through mating-type switching [61]. By combining morphological observations of the typical conidial structure with phylogenetic analyses, we achieved the identification of the novel fungal taxon *H. hongkongensis*. The inclusion of light (LED) for incubation, as well as the use of several marine-sourced natural substrates for cultivation, enabled the conidiation of the analyzed *H. hongkongensis* strains. We utilized the woods of a mangrove tree *A. corniculatum* within the vicinity of the studied seagrass meadow as substrates for cultivating *H. hongkongensis*, which were also considered ideal substrates for our Lulworthiaceae fungal isolates, in addition to the host seagrass *H. ovalis* tissues, given that Lulworthiaceae species have frequently been reported from marine woody substrates [5]. The limited growth of *H. hongkongensis* on terrestrial banana leaves [13] in contrast to its active expansion on mangrove tree and seagrass tissues may suggest the good adaptation of Lulworthiaceae to marine-associated substrates and its halophilic properties.

## 5. Conclusions

The present study provides the first thorough investigation on the largely unknown diversity and ecology of seagrass-associated fungi in Hong Kong by synergistically employing both culture-based methods and high-throughput sequencing. The novel fungal species assigned to a novel genus *H. hongkongensis* isolated from *H. ovalis* roots and rhizomes represents the first novel Lulworthiaceae taxon in Hong Kong, expanding our knowledge on the distribution and systematics of the obligate marine fungal family Lulworthiaceae. Additionally, this discovery highlights *H. ovalis* as a new seagrass host for Lulworthiaceae fungi. Introducing light and marine-linked culture substrates, including seagrass and mangrove tree tissues, effectively improved the conidiation of *H. hongkongensis* and enabled a better illustration of its morphological traits. ITS2 high-throughput sequencing allowed for the screening of *H. hongkongensis* distributed in different niche types. The results demonstrate a specialized and enduring relationship between *H. hongkongensis* and the host *H. ovalis*, with the former exhibiting a remarkable preference for colonizing the root and rhizome internal tissues of the latter, despite pronounced changes in environmental conditions in different periods of the year. The inhibitive effect exhibited by *H. hongkongensis* against Gram-positive *S. aureus* SA29213 and ATCC 43300 (methicillin-resistant) suggests its potential for producing antibacterial compounds, thereby rendering it a promising candidate for marine natural product discovery. Our study represents a crucial starting point in unraveling the obscure role of fungal partners residing in marine plants.

## Figures and Tables

**Table 1:** Endophytic fungi from *Halophila ovalis* roots and rhizomes with their GenBank accession numbers and BLAST matches based on ITS, SSU, and LSU sequences.

Isolate ID	Isolation Source	ITS		SSU		LSU	
		GenBank Code	Best Match(es) Information	GenBank Code	Best Match(es) Information	GenBank Code	Best Match(es) Information
HOMAR1 to HOMAR10 (10 strains)	Roots and rhizomes	PP350734–PP350743	All isolates matched with *Lulwoana uniseptata* strain Cs/1/10/1S3 (JQ801457.1), with identity ranging from 99.81% to 100%.	PP347844–PP347853	All isolates matched with *Lulwoana uniseptata* strain CBS 167.60 (AY879034.1), with identity of 99.45%.	PP347858–PP347867	All isolates matched with Lulworthiaceae sp. strain SLF 0120.1411 and strain SLF 0117.0203 (OR672790.1 and OR672789.1), with identity ranging from 98.63% to 98.77%.
			Seo et al. 2012 [50]		Campbell et al. 2005 [9]		Unpublished

In Isolate ID, HO indicates the initial letters of the seagrass host *Halophila ovalis* and MAR represents the month in which the sample was collected. The Arabic numerals indicate the isolated strains.

## Data Availability

The fungal DNA sequences amplified during this study are available in GenBank under accession numbers PP347844–PP347853 (SSU sequences), PP347858–PP347867 (LSU sequences), and PP350734–PP350759 (ITS sequences), and in the Sequences Read Archive of NCBI as BioProject ID PRJNA1062533.

## Supplementary Materials

The following supporting information can be downloaded at: https://www.mdpi.com/article/10.3390/jof10070474/s1, Table S1. Fungal isolates from *Halophila ovalis* rhizosphere soil with their GenBank accession numbers and BLAST matches based on ITS sequences. Table S2. Inhibition zones of fungal isolates from *Halophila ovalis* rhizosphere soil, rhizome, and root against pathogenic bacteria *Staphylococcus aureus*, *Escherichia coli,* and *Klebsiella pneumoniae*. Table S3. Abundance of operational taxonomic units (OTUs) in each sample. Table S4. Taxonomic information of operational taxonomic units (OTUs). Table S5. Abundance of OTUs classified as “*Lulwoana*” at genus level in each sample. Figure S1. Colony and microscopic features of fungal isolates belonging to twelve species isolated from seagrass *Halophila ovalis* rhizosphere soil. Ten-day-old colonies at 25 °C on PDA and slide cultures at 5 days were used for observation. (A) *Penicillium citrinum*; (B) *Aspergillus oryzae*; (C) *Aspergillus aculeatus*; (D) *Trichoderma harzianum*; (E) *Nectria* sp.; (F) *Aspergillus tubingensis*; (G) *Aspergillus westerdijkiae*; (H) *Penicillium mexicanum*; (I) *Penicillium steckii*; (J) *Penicillium subrubescens*; (K) *Penicillium brefeldianum*; (L) *Coprinellus disseminates*. Isolate ID is shown in each subfigure. Scale bar: 10 μm. Figure S2. Phylogenetic tree derived from maximum likelihood analysis based on ITS dataset constructed in IQ-TREE, with 1000 ultrafast bootstraps using TIM2e+G4 model selected by ProtTest. ITS2 sequence of representative OTU (ID: 1061269a449f32ed85148edd5cefe57e) obtained from amplicon sequencing classified as “*Lulwoana uniseptata*” based on UNITE Database v9.0 was also included for tree construction. Bootstrap (BS) values (>50) from maximum likelihood analysis followed by posterior probability (PP) values (>0.50) from Bayesian analysis are added to left of each node (BS/PP). Tree is rooted on Pleosporales species.
