# Supplementary material for: Halophilomyces hongkongensis, a Novel Species and Genus in the Lulworthiaceae with Antibacterial Potential, Colonizing the Roots and Rhizomes of the Seagrass Halophila ovalis
Source: J Fungi (Basel). 2024 Jul 10;10(7):474. doi: 10.3390/jof10070474 (PMC11278098; doi:10.3390/jof10070474)
Supplement: Supplementary file 1 [file jof-10-00474-s001.zip › Table S1.pdf]

Table S1. Fungal isolates from *Halophila ovalis* rhizosphere soil with their GenBank Accession Numbers and BLAST matches based on ITS sequences.

Strain ID	GenBank Code	BLASTn Search			
		Best Match(es)	Reference Accession Code	Overlap length	Identity (%)
HOMAR11	PP350744	*Penicillium citrinum*	JN851020.1	933	99.80
HOMAR12	PP350745	*Penicillium subrubescens*	OM372818.1	981	99.45
HOMAR13	PP350746	*Aspergillus oryzae*	MK748312.1	1000	99.46
HOMAR14	PP350747	*Penicillium citrinum*	MH059536.1	922	99.80
HOMAR15	PP350748	*Aspergillus aculeatus*	KM520043.1	974	99.81
HOMAR16	PP350749	*Trichoderma harzianum*	MZ681937.1	1055	99.83
HOMAR17	PP350750	*Nectria* sp.	MN701668.1	928	99.61
HOMAR18	PP350751	*Coprinellus disseminatus*	OP735466.1	1188	99.69
HOMAR19	PP350752	*Aspergillus tubingensis*	MT079171.1	1024	99.82
HOMAR20	PP350753	*Aspergillus westerdijkiae*	KP278178.1	957	99.62
HOMAR21	PP350754	*Penicillium* sp.	KX067851.1	970	99.81
		Penicillium mexicanum	OQ048471.1	970	99.81
HOMAR22	PP350755	*Trichoderma* sp.	MW940741.1	1029	100
		Trichoderma harzianum	KT783517.1	1026	100
HOMAR23	PP350756	*Penicillium citrinum*	OM279522.1	926	99.61
HOMAR24	PP350757	*Trichoderma harzianum*	MK377316.1	1050	99.65
HOMAR25	PP350758	*Penicillium* sp.	OK180504.1	994	100
		Penicillium steckii	OK605032.1	987	99.81
HOMAR26	PP350759	Fungal sp.	MF375408.1	1002	99.28
		Penicillium brefeldianum	KM243921.1	992	99.63

In isolate ID, HO indicates the initial letters of the seagrass species *Halophila ovalis*, MAR represents the month in which the sample was collected. The last Arabic numerals the isolated strains.
