# Supplementary material for: Halophilomyces hongkongensis, a Novel Species and Genus in the Lulworthiaceae with Antibacterial Potential, Colonizing the Roots and Rhizomes of the Seagrass Halophila ovalis
Source: J Fungi (Basel). 2024 Jul 10;10(7):474. doi: 10.3390/jof10070474 (PMC11278098; doi:10.3390/jof10070474)
Supplement: Supplementary file 1 [file jof-10-00474-s001.zip › Table S2.pdf]

Table S2. Inhibition zones of fungal isolates from *Halophila ovalis* rhizosphere soil, rhizome and root against pathogenic bacteria *Staphylococcus aureus*, *Escherichia coli* and *Klebsiella pneumoniae*.

ID	Fungal Identification	Gram-Positive		Gram-Negative	
		S. aureus SA29213	*S. aureus* (MRSA) ATCC 43300	*E. coli* ATCC 25922	*K. pneumoniae* Hvkp2
Antibiotic (Control)	-	34.75 (Vancomycin)	33.25 (Vancomycin)	31.33 (Colistin)	42 (Tigecycline)
HOMAR4	*Halophilomyces hongkongensis*	16	14.67	-	-
HOMAR5	*Halophilomyces hongkongensis*	11	12.5	-	-
HOMAR7	*Halophilomyces hongkongensis*	16.33	14.33	-	-
HOMAR8	*Halophilomyces hongkongensis*	10.5	11.5		
HOMAR11	*Penicillium citrinum*	17	16.33	-	-
HOMAR12	*Penicillium subrubescens*	-	-	-	-
HOMAR13	*Aspergillus oryzae*	-	-	-	-
HOMAR14	*Penicillium citrinum*	21.5	19	-	-
HOMAR15	*Aspergillus aculeatus*	10.33	9	-	-
HOMAR16	*Trichoderma harzianum*	10.67	10.67	-	-
HOMAR17	*Nectria* sp.	23.67	29.33	-	-
HOMAR18	*Coprinellus disseminatus*	-	-	-	-
HOMAR19	*Aspergillus tubingensis*	-	-	-	-
HOMAR20	*Aspergillus westerdijkiae*	15.67	10.33	21.33	15
HOMAR21	*Penicillium mexicanum*	-	-	11.67	-
HOMAR22	*Trichoderma harzianum*	17	15.67	-	-
HOMAR23	*Penicillium citrinum*	16.33	-	-	-
HOMAR24	*Trichoderma harzianum*	11	9.67	-	-
HOMAR25	*Penicillium steckii*	-	-	-	-
HOMAR26	*Penicillium brefeldianum*	-	-	-	-

The values show the average inhibition zone diameters in millimeter (mm).
