# Supplementary figures and images for: Halophilomyces hongkongensis, a Novel Species and Genus in the Lulworthiaceae with Antibacterial Potential, Colonizing the Roots and Rhizomes of the Seagrass Halophila ovalis
Source: J Fungi (Basel). 2024 Jul 10;10(7):474. doi: 10.3390/jof10070474 (PMC11278098; doi:10.3390/jof10070474)

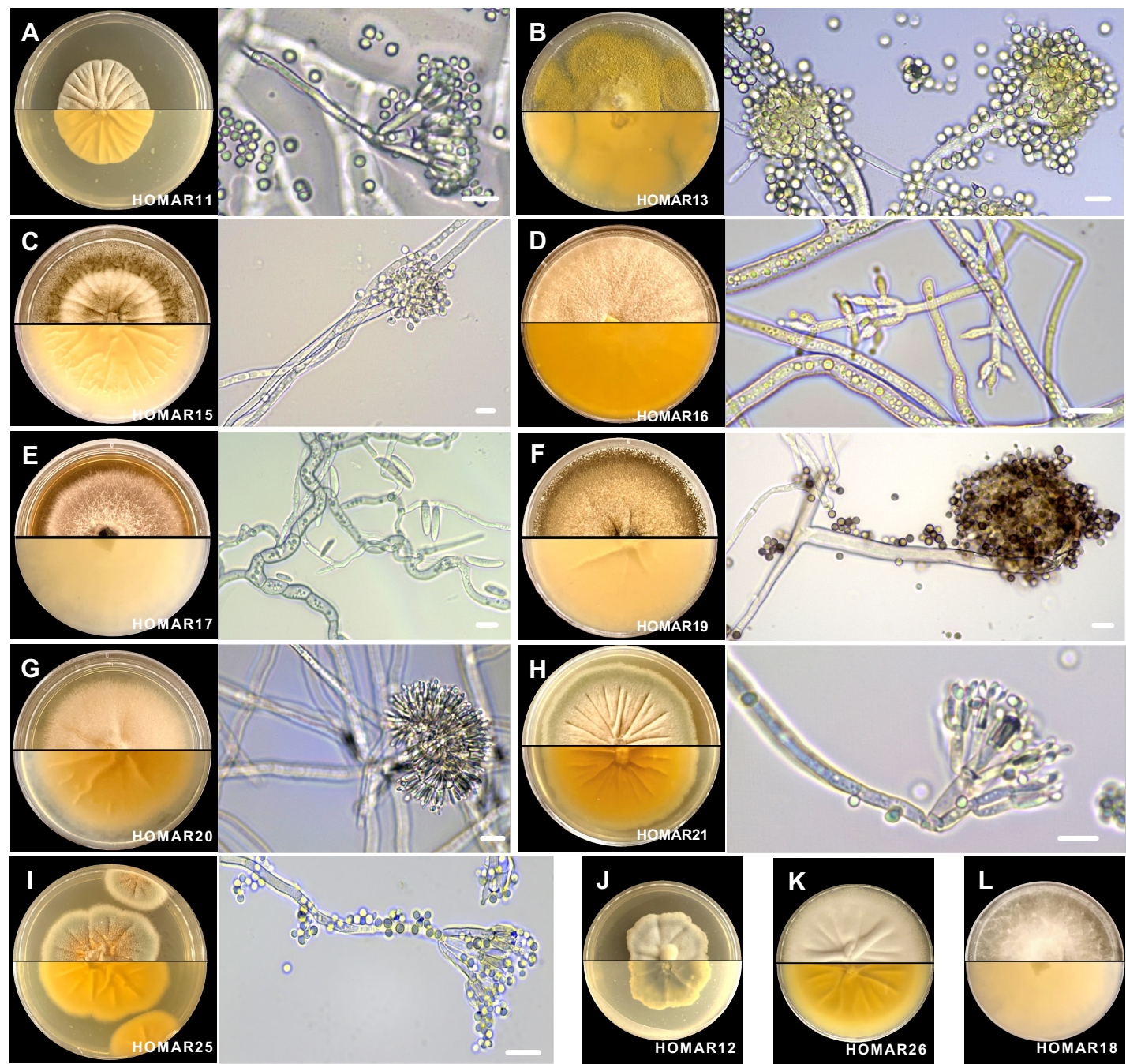

Supplement: Supplementary file 1 [file jof-10-00474-s001.zip › Figure S1.pdf]

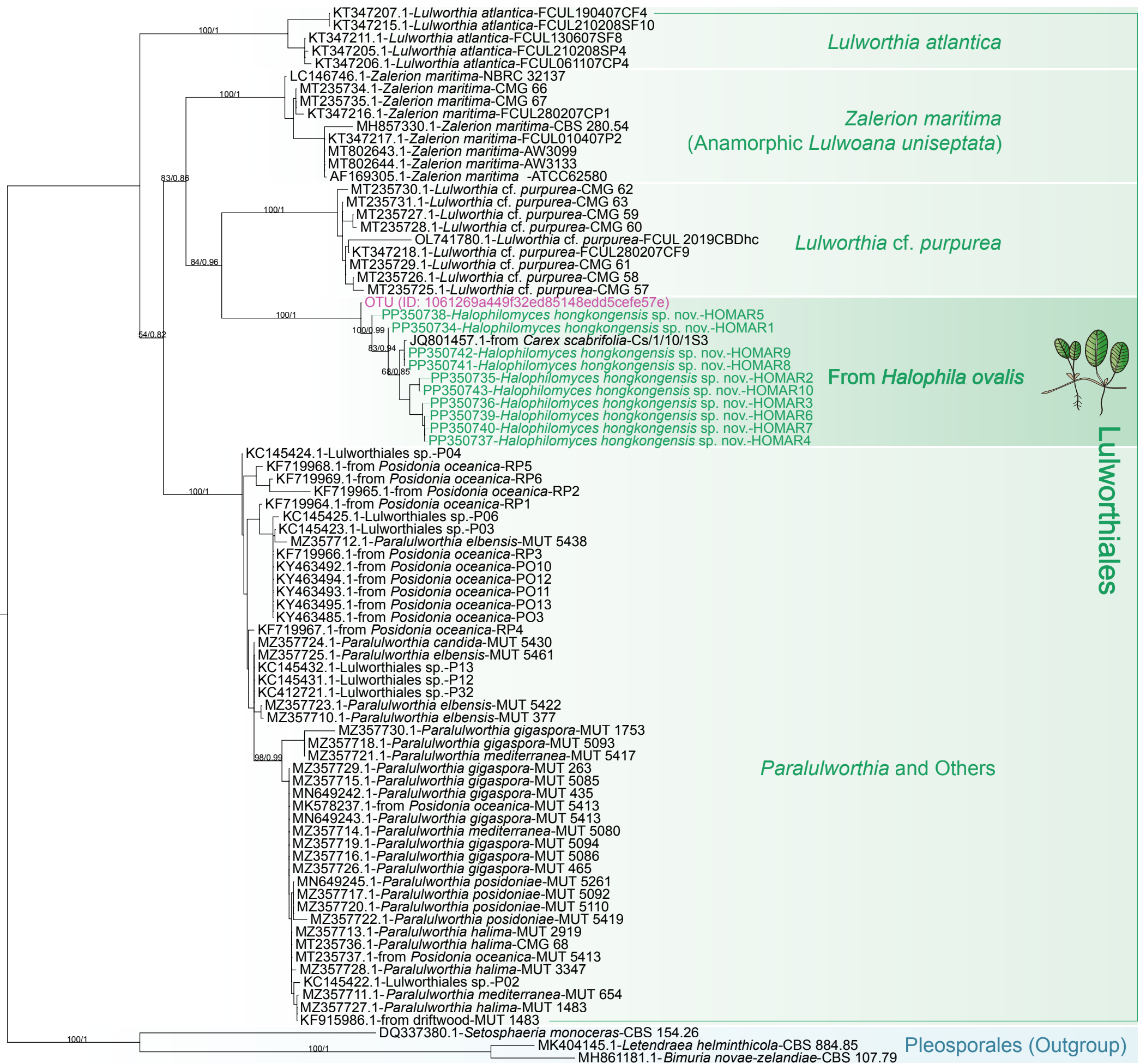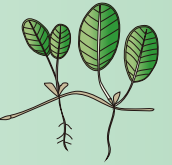

Lulworthiales

Supplement: Supplementary file 1 [file jof-10-00474-s001.zip › Figure S2.pdf]
